# To Buy or Not to Buy? Consumer Attitudes and Purchase Intentions for Suboptimal Food

**DOI:** 10.3390/ijerph15071431

**Published:** 2018-07-06

**Authors:** Song-Lin Wong, Cheng-Chin Hsu, Han-Shen Chen

**Affiliations:** 1 Department of Nutrition, Chung Shan Medical University, No. 110, Sec. 1, Jianguo N. Rd., Taichung City 40201, Taiwan; sonydiet@gmail.com (S.-L.W.); king@csmu.edu.tw (C.-C.H.); 2 Department of Health Diet and Industry Management, Chung Shan Medical University, No. 110, Sec. 1, Jianguo N. Rd., Taichung City 40201, Taiwan; 3 Department of Medical Management, Chung Shan Medical University Hospital, No. 110, Sec. 1, Jianguo N. Rd., Taichung City 40201, Taiwan

**Keywords:** extended theory of planned behavior (TPB), environmental concern, sensory appeal, food security

## Abstract

Food system and food safety have drawn spontaneous global attention due to the effect of substantial environmental concerns. Three billion tons of food are wasted every year, estimated as being a third of all produced food. The production of much of this waste is directly linked to the unwillingness to sell, purchase, and consume suboptimal food that have deviated from regular products in terms of appearance standards, date labeling, or damaged packaging. Yet empirical research on this issue is scarce. This study aims to develop an extended Theory of Planned Behavior (TPB) research model, which includes environmental concern and sensory appeal to predict consumers’ purchase intention to suboptimal foods. A total of 539 respondents collected in Taiwan as data input. The empirical results of structural equation modeling (SEM) indicate that consumers’ attitude was the main predictor of their intention to purchase suboptimal foods. Interestingly, this research showed that both perceived behavioral control and subjective norms were not significant predictors of intention. Furthermore, adding environmental concern and sensory appeal as the additional constructs to the TPB significantly increased the explanatory power of the standard model. These findings provide important insights for suboptimal food and useful recommendations for marketing channels, suggesting that promotion of suboptimal food may be the key to potential business.

## 1. Introduction

According to the Food and Agriculture Organization of the United Nations (FAO) research data [1], at least one-third of the world’s food is wasted every year. The total amount is almost as much as three billion tons. Lebersorger and Schneider [2] discovered in a study in Austria that food waste has increased since July 2014, reaching a peak in December 2014. Bilska et al. [3] indicates that the causes of food waste at the supply chain include such factors as incorrect package labels, incorrect food product weight labels, damaged expiration date labels and packaging, among others. In addition, according to the Taiwan Food and Drug Administration [4] report, Taiwan throws out 3.6 thousand tons of food a year. Nearly thirty percent of this amount is suboptimal food thrown directly in the trash. Hooge et al. [5] show that even though suboptimal foods differ from regular products in appearance, expiration date or packaging, their quality and safety are similar. Consumers often reject edible foods with changes in visual, sensory quality, or which passed the expiration date [6]. Therefore, the World Food Programme (WFP) is advocating the Food Recovery plan. The goal is to create a three-way benefit for society, economy and the environment through food waste reduction [7,8]. However, there is not much related research focusing on suboptimal food, which is the reason for this study.

A number of models, designed to better predict and understand human behaviors, have been proposed by social psychologists over the past several decades. Among these models, the theory of planned behavior (TPB) is the one that is most frequently utilized, and it is widely regarded as being effective when used for predicting behavior in general [9,10,11]. For this reason, a range of researchers have successfully utilized the TPB in order to better understand consumer decisions regarding food, a phenomenon which explains, in turn, why consumer food choices have become a topic of substantial interest among researchers conducting TPB-related studies [12,13,14,15,16,17,18,19].

**Figure 1:**
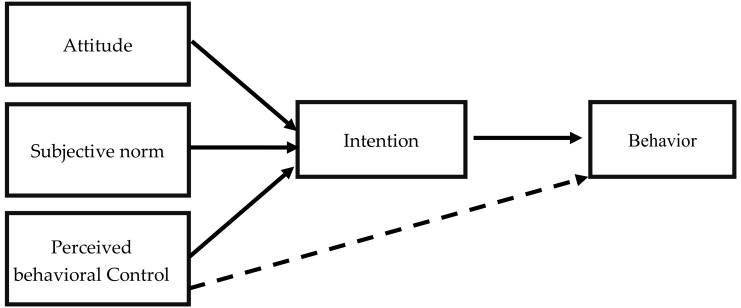
Theory of planned behavior.

According to the TPB, a combination of three factors is responsible for the formation of behavioral intentions, with those factors being subjective norms, perceived behavioral control, and attitudes toward the behavior in question [20]. Figure 1 depicts the theory in the form of a structural diagram. In this context, an attitude toward a behavior is defined as the ‘degree to which a person has a favorable or unfavorable evaluation or appraisal of the behavior in question’. Three main components in the attitude of a person, including affect, cognition and behavior. The first component is affect. This component relates to a person’s emotional response to product. The second component is cognition. This component is a person’s belief or knowledge about product. The latter component is behavior. This component relates to the tendency of a person to perform a certain action with regard to its attitude towards product. While a subjective norm refers to any ‘perceived social pressure to perform or not perform the behavior’. Finally, perceived behavioral control refers to ‘an individual’s perceived ease or difficulty of performing the particular behavior’. Accordingly, if an individual perceives himself or herself to have a relatively high level of behavioral control with respect to a given behavior, then that individual will have a greater likelihood of exhibiting a firm intention to engage in said behavior. The term behavioral intention itself, meanwhile, refers to the individual’s willingness to engage in the behavior in question, with the assumption being that such willingness must necessarily exist immediately prior to the actual behavior itself being carried out [20].

According to Ajzen [20], the TPB can be modified through the addition of new variables or by changing the path for existing variables. Relatedly, previous studies have found that, while the TPB fundamentally assumes that behavioral intentions are the result of subjective norms, attitudes, and perceived behavioral control, a number of domain-specific factors not incorporated in the model are also relevant [21,22]. Moreover, recent studies in the field of psychology have generated noteworthy evidence supporting the inclusion of various predictor variables in the TPB. More specifically, the value of the TPB in terms of its predictive power regarding various domains has been shown to be enhanced when additional predictor variables are included [22,23]. The study has also attempted to include new constructs (environmental concern and sensory appeal) in the TPB taking support from the extant literature.

When consumers purchase products, they do not base their decisions purely on their preferences for the product. Consumer behavioral changes that arose from the increase in environmental awareness include: valuing a low-carbon diet, encouraging support for local produce, supporting the purchase of seasonal, organic and fair-trade products, and attaching greater importance to product labeling [24,25]. Aschemann-Witzel et al. [26] explored consumer behavior towards price-reduced suboptimal foods in the supermarket and the relation to food waste in households. Moreover, Loebnitz and Grunert [27] also discovered that food abnormality, consumers’ environmental concerns and social trust are significantly correlated with product purchase desire. Aschemann-Witzel [28] explored the factors that influence acceptance of expiration date based pricing of suboptimal food by applying four sets of stickers writing “fight foodwaste”, “lower price”, “save more” and “fight foodwaste and lower price- save more” to the suboptimal food items in store. Therefore, this study will consider environmental concern as one of the research variables.

Baker et al. [29] indicates that a product’s sensory appeal will affect consumers’ product choice preference and purchase desire. Sensory appeal refers to the appeal of the product’s taste, appearance, texture and smell to consumers [15]. Loebnitz and Grunert [27] shows that consumers will choose fruits and vegetables with a perfect appearance. Thus, sensory appeal and purchase desire are inseparable. Symmank et al. [30] demonstrate a positive relationship between sensory perception, overall liking, and purchase intention for visually suboptimal bananas, yet overall liking and purchase intention decreases when the product exceeds a certain ripening status. Loebnitz and Grunert [31] indicated that consumers perceive abnormally-shaped vegetables as more risky, and paradoxically, they associate natural vegetable shape-abnormalities with GM, despite having no other information available. Hence, this study will also consider sensory appeal as one of the research variables.

Above all, this study utilizes the TPB model and considers the two research dimensions of environmental concern and sensory appeal. We aim to develop an extended Theory of Planned Behavior (TPB) model, which includes environmental concern and sensory appeal to predict consumers’ purchase intention to suboptimal foods. We hope that the findings will provide important insights for suboptimal food and useful recommendations for marketing channels, suggesting promotion of suboptimal food may be the key of potential business. 

## 2. Materials and Methods

### 2.1. Research Framework

**Figure 2:**
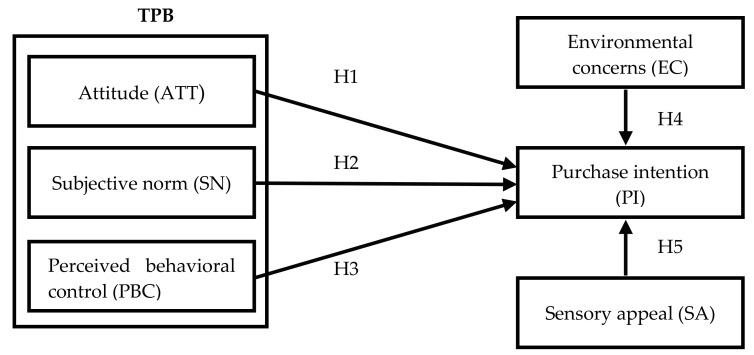
Conceptual framework and hypotheses of the study. H_1_. ATT will positively affect PI; H_2_. SN will positively affect PI; H_3_. PBC will positively affect PI; H_4_. EC will positively affect PI; H_5_. SA will positively affect PI.

TPB is the core of this study, incorporating the two facets of “environmental concern” and “sensory appeal” to form an extended TPB model. This study discusses Taiwanese people’s perceptions and views on suboptimal foods and further investigates their purchase desire. The proposed theoretical framework is shown in Figure 2.

### 2.2. Research Hypotheses

The relationship between attitudes and behaviors has previously been explored in various studies [32,33,34,35], with those studies having found that, in the context of green consumption settings, an attitude-intention rationale is dominant. Meanwhile, numerous other studies have found that, in the context of marketing and consumer behaviors, subjective norms serve as major factors in deciding people’s intentions, including their participation intentions [36], technology-use intentions [37], intentions to purchase organic foods [33,34], and intentions to revisit green hotels [38,39,40]. Relatedly, various investigations have demonstrated a positive relationship between intentions and perceived behavioral control in a range of research contexts, such as those involving intentions relating to recycling [41], conservation [42], green hotels [38,39,40,43], organic foods [44,45], and green products in general [46]. Our literature review suggests that a shift in attitude, subjective norm and perceived behavioral control towards suboptimal food purchase would increase the purchase intention for suboptimal food. Hypotheses for the standard TPB constructs.

**Hypothesis** **(H1).** Attitude will positively affect purchase intention of suboptimal food.

**Hypothesis** **(H2).** Subjective norm will positively affect purchase intention of suboptimal food.

**Hypothesis** **(H3).** Perceived behavioral control will positively affect purchase intention of suboptimal food.

According to a previous study by Dunlap and Jones [47], the term environmental concern refers to ‘the degree to which people are aware of problems regarding the environment and support efforts to solve them or indicate the willingness to contribute personally to their solution’. Such concern for the environment on the part of an individual, in addition to being a topic of critical importance to environmental research in general, has a clear and substantial relationship with any environmentally friendly behaviors that the individual engages in [48]. For example, a previous study by Pagiaslis and Krontalis [49] found that a consumer’s intention to buy ecofriendly products is positively and directly affected by that consumer’s level of environmental concern. Likewise, the intention to purchase organic foods has been reported to be significantly impacted by environmental concern [50]. At the same time, another study found that those consumers who exhibit a preference for organic products also show a greater inclination for involvement in eco-friendly activities, a propensity which, in turn, demonstrates their environmental concern [51]. More specifically, one way in which relatively high levels of environmental concern may be demonstrated is via increased levels of consumption of suboptimal food. The discussion results into the following hypotheses:

**Hypothesis** **(H4).** Environmental concerns will positively affect purchase intention of suboptimal food.

Taste, smell, and appearance are generally considered the sensory attributes of food, and when choosing which foods to consume, such sensory attributes have long been known to constitute one of the most critical factors that consumers consider [52]. Unsurprisingly, such sensory characteristics have likewise been found to be a significant motive in determining purchases of organic foods [53]. Some studies revealed that sensory attributes of organic food such as taste, color, and texture are linked with purchase intention [54,55]. Accordingly, we hypothesize that:

**Hypothesis** **(H5).** *Sensory appeal will positively affect purchase intention of suboptimal food*.

### 2.3. Questionnaire Design

The questionnaire for the study was designed by adopting items from relevant literature. Items were measured using a 7 point Likert’s scale, where 7 indicates a positive view (Strongly Agree) and 1 represents a negative view (Strongly Disagree). The questionnaire items and their source of adoption are mentioned in Table 1.

### 2.4. Sample Size and Composition 

The sample size required for this study was computed based on Hair et al., [60] recommendation of a desired level of 15–20 observations per studied variable. Our study has six constructs (4 items for Attitude (ATT), 3 items for Subjective Norm (SN), 3 items for Perceived Behavioral Control (PBC), 4 items for Environmental Concern (EC), 3 items for Sensory Appeal (SA) and 4 items for purchase intention (PI), totaling 21 items) resulting into ideal sample size of 420 (=20 × 21) respondents. However, 539 responses were considered for analysis, which was higher than the commended value of at least 400 for structural equation modeling (SEM) [61]. 

From descriptive statistics, Table 2 summarized that majority of the respondents in sample are female, married, with a family size of 2–4 persons, and a monthly income higher than TWD 40,000 per person. Most of the sample fell in the 36–50 age group.

### 2.5.Statistical Analysis

The theoretical framework was analyzed using SPSS (Statistical Package for Social Science, IBM Corp.: New York, NY, USA) and AMOS (Analysis of Moment Structure, IBM Corp.: New York, NY, USA) version 21 [62]. Two SEM study models were investigated in a study [60]. The two models, namely a measurement model and a structural model, were used to test for validity and reliability, and to test for model fit and hypothesis testing, respectively.

## 3. Results

### 3.1. Measurement Model: Reliability and Validity

The measurement Model provides the quantitative measures of the validity and reliability for the constructs. Using Cronbach’s α, internal consistency among the items was measured, the score ranges from 0.761 to 0.890 which lies between the acceptable limit of 0.7 and higher [60]. Further, the convergent and discriminant validity were measured. Convergent validity was measured on the basis of three components: composite reliability (CR), factor loading and Average Variance Extracted (AVE). The value of composite reliability ranged from 0.705 to 0.912 which implies that all constructs met the recommended criterion of 0.6 and higher [63]. The value of factor loading (0.703–0.836) was higher than the recommended level of 0.6 [64]. The value of A.V.E ranged from 0.623 to 0.750, which also met the acceptable lower limit of 0.5 [60]. The detail of reliability and convergent validity are outlined in Table 3. Means, standard deviations, and correlations among the constructs are presented in Table 4.

### 3.2. Structural Model: Goodness of Fit Statistics and Hypothesis Testing

Further, the theoretical framework was tested for goodness of fit indices. The results showed that goodness of fit statistics of the theoretical framework represents a good fit, as it lies in the acceptable limit (*χ*^2^ = 203.653, *χ*^2^/df = 1.182, Goodness of Fit index(GFI) = 0.935, Tucker Lewis Index (TLI) = 0.981, Comparative Fit Index (CFI) = 0.986, Incremental Fit Index (IFI) = 0.988, Root Mean Square Error of Approximation (RMSEA) = 0.032). The observed value of Adjusted Goodness of fit index (AGFI) was 0.891 that exceeds the cutoff level of 0.8 [65]. All other fit indices were above the recommended criteria [63]. Taken together, the above results indicate that the proposed theoretical framework fits the data. Finally, a comparison between the TPB model and the proposed theoretical framework was also conducted, and the results of that comparison indicate that the framework has a better model fit (adjusted R^2^ = 0.586) than the TPB (adjusted R^2^ = 0.402) in terms of measuring the intentions of consumers regarding the purchase of suboptimal food. As a result, all indices provided evidence of an acceptable measurement model. (Table 5).

### 3.3. Results of SEM

**Figure 3:**
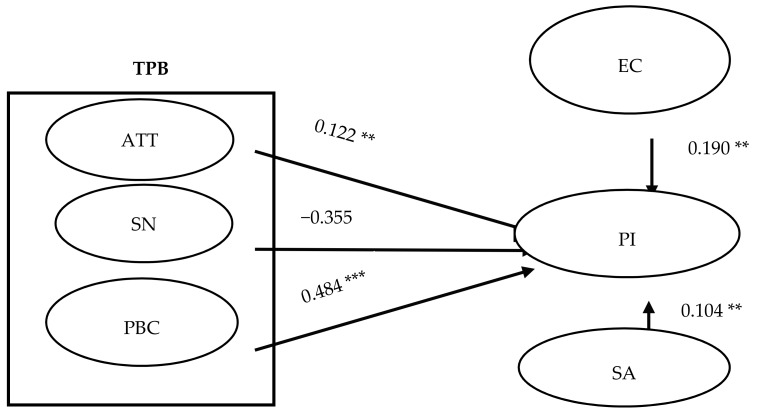
Results of SEM. ** *p* < 0.01; *** *p* < 0.001; GFI = 0.935; AGFI = 0.891; TLI = 0.981; CFI = 0.986; RMSEA = 0.032.

The results of the participants’ purchase intention concerning suboptimal food are shown in Figure 3. Attitude (ATT) had a positive influence on purchase intention (PI) (β = 0.122, *p* < 0.01), and Perceived Behavioral Control (PBC) had a positive significant influence on purchase intention (PI) (β = 0.484, *p* < 0.001). Environmental Concern (EC) had a positive significant influence on purchase intention (PI) (β = 0.190, *p* < 0.01). Sensory Appeal (SA) had a positive significant influence on purchase intention (PI) (β = 0.104, *p* < 0.01). However, Subjective Norm (SN) had a negative influence on purchase intention (PI) (β = −0.355, *p* > 0.05). Based on these findings, H1, H3, H4, and H5 are supported, but H2 are not.

## 4. Discussion

A summary of the verification of the hypotheses made in this study is shown in Table 6. In this study, the clear identification of the determining factors of consumer attitudes and intentions regarding the purchase of suboptimal foods was the main research objective. To this end, the TPB was utilized as the theoretical framework for the study, while the study also sought to expand upon the TPB via the incorporation of additional constructs within it. At first, H1 and H3 were supported. The results of the SEM showed that it was important whether purchasing suboptimal foods would be desirable (attitude) or easy to purchase (perceived behavioral control). The results of the study indicated that consumers’ intentions toward the purchase of suboptimal foods are significantly affected by the attitudes of those consumers toward the suboptimal food in question, as well as by the consumers’ perceived behavioral control, which is similar to conclusions drawn in Dean et al. [33] and Zhou et al. [35]. On the other hand, subjective norms of suboptimal foods did not directly affect purchase intention. Therefore, we concluded that H2 was not supported, whereas the subjective norms of the consumers were not found to exhibit any significant effects on said purchase intentions.

More specifically, the results indicated that, among the key variables of the TPB, perceived behavioral control is the factor most responsible for determining the intentions of consumers regarding the purchase of suboptimal food, a finding which in turn suggests that consumers deal in a relatively effective manner with factors which could be regarded as disabling factors. That said, it should be reiterated that the purchase intentions of consumers regarding suboptimal food were found to also be affected to a significant extent by their attitudes towards the suboptimal food in question. This finding underscores the substantial relevance of consumers having positive attitudes regarding a given suboptimal food when they are engaged in the process of considering whether or not to buy it. Meanwhile, because the subjective norms of the consumers in this study were not found to exhibit any significant influence on the intentions of those consumers toward the purchase of suboptimal foods, it can be concluded that that the purchasing of suboptimal foods has not yet become a social norm among people in Taiwan. 

The additional constructs incorporated in the TPB model in this study were environmental concern and sensory appeal. The research hypothesis (H4) states that the more consumers are concerned about the environment, the more likely they are to buy suboptimal foods. The results indicated that environmental concern had significant effect on the intentions of the consumers in this study to purchase suboptimal foods. The results of this study are consistent with research by Pagiaslis and Krontalis [49], and Smith and Paladino [50]. It proves that consumers in Taiwan now do care much about environmental issues.

At last, H5 was supported. the study results indicated that the respondents’ purchase intentions were significantly and positively affected by their perceptions of the sensory appeal of suboptimal food. The results of this study are consistent with research by Fotopoulos et al. [54], and Padel and Foster [55]. In other words, in the event that consumers have the perception that buying a suboptimal food is exciting, fun, or otherwise pleasurable in some regard, the sensory experience conveyed by the food will be a relevant factor in determining the consumers’ purchase intentions toward the food. 

The results of the study indicated that both the original TPB and the proposed theoretical framework fit the data well. However, the model fit of the TPB in terms of the explained variance (that is, the adjusted R^2^) was substantially enhanced by the incorporation of the additional constructs of environmental concern and sensory appeal within the TPB as part of the proposed framework, with the explained variance increasing from 40.2% for the original TPB to 58.6% for the proposed framework. This boost to the model fit validates the incorporation of these two constructs within the TPB, at least when utilizing the TPB for the purpose of predicting the intentions of consumers in a developed nation regarding the purchase of suboptimal foods.

According to the questionnaire results, any lack of access to suboptimal foods poses a substantial barrier to consumers. More specifically, the results indicated that consumers take a practical approach toward the purchase of food and do not want to make visits to several stores in order to find the products they want. As such, when developing a strategy for the sale or marketing of suboptimal foods, the availability of the foods must be regarded as a particularly important parameter to consider. In general, the study results indicated that consumers exhibit high intentions to purchase suboptimal foods (mean of PI = 6.12/7). As such, the interested parties (such as farmers, wholesalers, etc.) should strive to enhance the distribution channels for suboptimal foods so that the foods will be available at markets and stores that are convenient for their intended customers, thus making it easy for those consumers to purchase the suboptimal foods.

## 5. Conclusions

The present study had two primary goals. First, the study sought to explore the utilization and value of the TPB when applied to investigating the intentions of consumers toward the purchase of suboptimal foods. Second, the study sought to enhance the predictive power of the TPB through the incorporation of two additional constructs within it, namely, sensory appeal and environmental concern. 

### 5.1. Conclusions

The results of the study indicated three key findings. The first is confirmation that, with respect to consumer intentions regarding the purchase of suboptimal foods, the TPB can serve effectively as a framework for predicting said intentions. The second, however, is partial support for the notion that the utility of the TPB can be increased further still through the incorporation of metrics of sensory appeal and environmental concern. In other words, both sensory appeal and environmental concern appear to be useful constructs in furthering the understanding and predictability of consumer intentions regarding the purchase of suboptimal foods. Relatedly, the results of this study provide support for existing evidence regarding the relevance of these two constructs in the prediction of intentions with an environmental orientation. Finally, the third key finding of the present study is that among consumers in Taiwan specifically, personal attitudes, sensory appeal, and environmental concern are all of critical importance in any effort to predict consumer intentions regarding the purchase of suboptimal foods. At the same time, further investigations will be necessary in order to further illuminate the precise mechanisms through which sensory appeal and environmental concern exert their effects on purchase intentions.

### 5.2. Limitations of the Research and Future Research

This study had a number of limitations. First, rather than being asked for their feelings regarding a specific category of products, the study respondents were queried regarding their broad perceptions of various attributes and their behavioral intentions toward suboptimal food in general. Relatedly, because the behaviors and expectations of consumers may vary with respect to different categories of suboptimal food products (such as dairy foods vs. non-dairy foods), it is suggested that future studies regarding the purchase of suboptimal foods could provide additional insights by investigating more domain-specific attitudes and purchase intentions, that is, by investigating such attitudes and intentions toward specific suboptimal food items or categories. Another limitation of the present study was that it did not attempt to investigate the influences of any moderating effects. As such, future investigations could build upon the findings of the present study by looking at the impacts of such moderating effects, such as, for example, the socio-demographic attributes of consumers and their levels of trust in various actors within the food sector (such as farmers, producers, and vendors). Furthermore, it is suggested that future studies could conduct comparisons of those consumers who prefer and do not prefer suboptimal foods in order to ascertain the differing characteristics of the two groups, if any, as well as exactly how their behaviors and perceptions differ with respect to both conventionally-produced and suboptimal foods.

## Figures and Tables

**Table 1:** Constructs/variables and corresponding measuring statements included in the questionnaire.

Construct/Variable	Number of Statements	Measuring Items	Sources of Adoption
Attitude (ATT)	4	Buying suboptimal food is a good idea. Buying suboptimal food is a wise choice. I like the idea of buying suboptimal food. Buying suboptimal food would be pleasant.	Wang et al. [56]
Subjective Norm (SN)	3	Most people, important to me, think that I should buy suboptimal food. Most people, important to me, would want me to purchase suboptimal food. People whose opinion I value would prefer that I should buy suboptimal food.	Han et al. [39]
Perceived Behavioral Control (PBC)	3	If I wanted to, I could buy suboptimal food instead of nonorganic food. I think it is easy for me to buy suboptimal food. It is mostly up to me whether or not to buy suboptimal food.	Ajzen [57] Arvola et al. [23]
Environmental Concern (EC)	4	The balance of nature is very delicate and can be easily upset. Human beings are severely abusing the environment. Humans must maintain the balance with nature in order to survive. Human interferences with nature often produce disastrous consequences.	Roberts & Bacon [58]
Sensory Appeal (SA)	3	Suboptimal food looks nice. Suboptimal food has a pleasant texture. Suboptimal food tastes good.	Steptoe et al. [52]
Purchase Intention (PI)	4	I am willing to consume suboptimal food if they are available for purchase I intend to consume suboptimal food if they are available for purchase I plan to consume suboptimal food if they are available for purchase I will try to consume suboptimal food if they are available for purchase	Ajzen [57] Arvola et al. [23] Yazdanpanah et al. [59]

**Table 2:** Sample characteristics.

Variable	Categories	Frequency	Percentage
Gender	Male	173	32.1
	Female	366	67.9
Age	Less than 20 years	21	3.9
	20–35 years	168	31.2
	36–50 years	287	53.2
	More than 50 years	63	11.7
Marital Status	Single	137	25.4
	Married	345	64.0
	Divorced/Widow	57	10.6
Family size	1 person	38	7.1
	2–4 persons	236	43.8
	5–6 persons	195	36.2
	More than 6 persons	70	12.9
Personal income monthly (TWD)	Less than 20,000	35	6.5
	20,001–40,000	166	30.8
	40,001–60,000	253	46.9
	More than 60,000	85	15.8

TWD = Taiwan Dollar.

**Table 3:** Results of factor loading, reliability and validity.

Constructs	Items	Factor Loading	Cronbach’s α	CR	AVE
Attitude (ATT)	ATT1	0.826	0.890	0.865	0.748
	ATT2	0.818			
	ATT3	0.832			
	ATT4	0.795			
Subjective Norm (SN)	SN1	0.790	0.884	0.912	0.750
	SN2	0.828			
	SN3	0.801			
Perceived Behavioral Control (PBC)	PBC1	0.703	0.809	0.821	0.623
	PBC2	0.836			
	PBC3	0.716			
Environmental Concern (EC)	EC1	0.762	0.761	0.705	0.673
	EC2	0.706			
	EC3	0.794			
	EC4	0.815			
Sensory Appeal (SA)	SA1	0.793	0.826	0.741	0.704
	SA2	0.825			
	SA3	0.807			
Purchase Intention (PI)	PI1	0.782	0.835	0.826	0.726
	PI2	0.797			
	PI3	0.804			
	PI4	0.816			

CR = Composite reliability, AVE = Average variance extracted.

**Table 4:** Means, standard deviations and correlations of constructs.

Construct	Mean	S.D.	1	2	3	4	5	6
1. Attitude (ATT)	6.05	0.83	1.00					
2. Subjective Norm (SN)	4.36	1.12	0.24	1.00				
3. Perceived Behavioral Control (PBC)	5.27	1.26	0.26	0.38	1.00			
4. Environmental Concern (EC)	5.41	1.05	0.42	0.37	0.35	1.00		
5. Sensory Appeal (SA)	5.26	1.16	0.51	0.48	0.33	0.41	1.00	
6. Purchase Intention (PI)	6.12	1.24	0.48	0.42	0.37	0.31	0.45	1.00

**Table 5:** Summary of Goodness-of Fit Indices for the Structural Models.

Model	*χ*^2^	*χ*^2^/df	GFI	TLI	RMSEA	IFI
Structural model	203.653	1.182	0.935	0.981	0.032	0.988
Recommended value	N/A	≥0.90	≥0.90	≥0.90	<0.08	≥0.90

**Table 6:** Summary of hypothesis verification.

Hypothesis	Content	Verification
H1	Attitude will positively affect purchase intention of suboptimal food.	Accepted
H2	Subjective norm will positively affect purchase intention of suboptimal food.	Rejected
H3	Perceived behavioral control will positively affect purchase intention of suboptimal food.	Accepted
H4	Environmental concerns will positively affect purchase intention of suboptimal food.	Accepted
H5	Sensory appeal will positively affect purchase intention of suboptimal food.	Accepted
